# Association of Hypertension and Obesity with Risk Factors of Cardiovascular Diseases in Children Aged 6–9 Years Old in the Eastern Cape Province of South Africa

**DOI:** 10.3390/children7040025

**Published:** 2020-03-28

**Authors:** Edna N. Matjuda, Godwill A. Engwa, Prescilla B. Letswalo, Muhau M. Mungamba, Constance R. Sewani-Rusike, Benedicta N. Nkeh-Chungag

**Affiliations:** 1 Department of Human Biology, Faculty of Health Sciences, Walter Sisulu University PBX1, Mthatha 5117, South Africa; engoakoana@gmail.com (E.N.M.); bpletswalo@gmail.com (P.B.L.); mmungamba@gmail.com (M.M.M.); crusike@wsu.ac.za (C.R.S.-R.); 2 Department of Biological and Environmental Sciences, Faculty of Natural Sciences, Walter Sisulu University PBX1, Mthatha 5117, South Africa; gengwa@wsu.ac.za

**Keywords:** hypertension, obesity, oxidative stress, cardiovascular disease, endothelial dysfunction, renal dysfunction

## Abstract

Cardiovascular disease (CVD) risk factors are known to begin early in life, but limited data on the relationship of obesity and hypertension with other known CVD risk factors, such as endothelial dysfunction, oxidative stress, and chronic low-grade inflammation is available on children. In this cross-sectional study involving 6–9 years old school children aged from the Eastern Cape Province of South Africa the relationship between obesity/hypertension and other risk factors for CVDs was investigated. General anthropometric parameters were measured, followed by blood pressure (BP) measurements and pulse wave velocity (PWV). Urine samples were collected for the determination of albumin, creatinine, asymmetric dimethylarginine (ADMA), 8-hydroxy-2deoxyguanosine (8-OHdG), and thiobarbituric acid-reactive substance (TBARS). Overweight/obesity (19.28%) and pre-hypertension/hypertension (42.16%) were prevalent in children. Mid-upper arm circumference (MUAC), a marker of obesity, was positively correlated with ADMA, while ADMA and PWV were significantly different (*p* < 0.05) between hypertensive and normotensive children. Also, TBARS and 8-OHdG were significantly (*p* < 0.05) increased in hypertensive subjects. Creatinine was significantly (*p* < 0.05) increased in obese, as well as in hypertensive children, and positively associated with waist circumference (WC) and neck circumference (NC). In conclusion, obesity and hypertension were associated with renal-cardiovascular disease risk, while oxidative stress showed a possible association with obesity in 6 to 9 year old South African children of African descent. This suggests that South African children of African descent may be becoming more prone to developing CVDs, and therefore may require early intervention for the prevention of CVDs in the near future.

## 1. Introduction

Cardiovascular diseases (CVDs) are the number one cause of death worldwide [1]. According to the World Health Organisation, approximately 17.5 million deaths, which account for about 31% of all death, were due to CVDs globally in 2012. Of this population, 80% occurred in developing countries [2]. CVDs are estimated to account for 18% of mortality in South Africa [3]. Though children are not part of these statistics, it is known that CVD risk factors begin early in life and tracks to adulthood [4].

South Africa is one of the countries undergoing rural to urban transition, and populations in this transition have been shown to experience an increase in the prevalence of overweight and obesity due to changes in diet, sedentary lifestyle, and stress [5]. The adoption of western diet contributes to the increasing rates of obesity and overweight in both children and adults in South Africa [6]. In obese individuals, adipocytes secrete fatty acids and release inflammatory factors, which lead to the development of atherosclerosis by promoting hyperlipidaemia, insulin resistance, oxidative stress, hypertension, and vascular inflammation [7]. Hypertension is one of the factors that are commonly associated with obesity [8,9] and one of the most important risk factors of CVDs [10]. Hypertension is known to be associated with endothelial dysfunction, as pressure and flow rate in the blood vessels affects smooth muscle tone [11,12].

Apart from the classical CVD risk factors, such as obesity, hypertension, and low high-density lipoprotein cholesterol which start in childhood and may persist into adulthood [13], other emerging CVD risk factors, such as endothelial dysfunction, oxidative stress, and low grade inflammation are now recognised as markers of subclinical atherosclerotic CVD [14]. Moreover, renal dysfunction markers, such as elevated creatinine and albumin, as well as the albumin-to-creatine ratio have been associated with risk of CVD in the adult population [15,16].

Obesity has been suggested to promote oxidative stress, as obesity promotes the infiltration and activation of macrophages and monocytes into adipose tissue [17] which in turn release elevated levels of pro-inflammatory adipokines, such as tumor necrosis factor alpha (TNF-a) and interleukin-6 (IL-6) [17,18]. These inflammatory markers induce the production of reactive oxygen species (ROS), thereby causing oxidative stress [19,20]. There are reports that oxidative stress may cause hypertension, as increased ROS may reduce NO levels by converting NO to more NO-reactive species [21]. A reduced level of NO leads to endothelial dysfunction and can impair endothelium-dependent vascular relaxation, thereby increasing vascular contractile activity, which may cause high blood pressure [21]. Oxidative stress has been associated with lipid, protein, and DNA damage. Thiobarbituric acid-reactive substances (TBARS) are commonly formed following cell membrane damage, while 8-hydroxyl-2-deoxy guanosine (8-OHdG) is commonly formed as a result of DNA damage due to oxidative stress [22].

Endothelial dysfunction has been established to be an early predictor, and also an initial factor to the progression of atherosclerotic cardiovascular disease [4]. Endothelial function of vasodilatation is mediated by nitric oxide (NO), which is the major contributor to endothelium-dependent relaxation in conduit arteries [23]. Impaired synthesis of NO leads to endothelial dysfunction. Asymmetric dimethylarginine (ADMA) is a molecule that can impair NO synthesis. ADMA inhibits endothelial nitric oxide synthase (eNOS) by competing with its substrate, L-arginine—thereby impairing the production of NO [24]. A high level of pulse wave velocity (PWV) is indicative of endothelial dysfunction, and thus, PWV has served as a marker for endothelial dysfunction.

CVDs include any disease of the blood vessels and the heart, such as coronary artery disease, stroke, arthrosclerosis, ischemic, and heart disease [2]. The combination of risk factors, such as hypertension and obesity, significantly increase the probability of CVD development [25]. A study conducted in Johannesburg found that the prevalence of hypertension was 19% in children aged 5–8 years old [26]. Moreover, a longitudinal study conducted in northwestern South Africa found that the overall obesity rate increased over a period of 3 years, from 12.5% at baseline to 16.7% among children aged 6–9 years old [27]. This indicates an increase in the incidence of childhood obesity. Although there have been concerns regarding the prevalence of hypertension and obesity in children around the world, there is limited data available on the relationship of obesity and hypertension with other risk factors of CVDs. Therefore, the present study aimed to assess the prevalence of obesity and hypertension and investigate their relationship with risk factors of CVDs in children.

## 2. Materials and Methods

### 2.1. Study Population and Design 

This was a cross-sectional study involving children aged 6–9 years old recruited from primary schools in Libode, Mthatha, and East London of the Eastern Cape Province of South Africa. All data were collected in school classrooms.

### 2.2. Ethical Consideration

Ethical clearance was obtained from the Health Sciences Ethics Committee of Walter Sisulu University, South Africa (Ref No: 112/2018 issued on 13 October 2018). After careful explanation of the purpose and aim of the study, written informed consent was obtained from the parents/legal guardians of the children before enrolment into the study. The study adhered to the standards of reporting and acted in accordance with the National Data Protection Acts, as the identities of the participants were kept confidential. There were no important changes to the methods after the study’s commencement.

### 2.3. Inclusion/Exclusion Criteria 

Children aged 6–9 years old of African ancestry and with no known cardiovascular and renal diseases were recruited for the study. Physically challenged children and those having any known comorbid or cardiovascular diseases, as well as children of non-African ancestry, were excluded from the study. 

### 2.4. Anthropometric Measurements 

Anthropometric measurements were performed in accordance with the International Standards for Anthropometric Assessments [28]. Participants’ heights were measured using a wall-mounted Harpenden stadiometer and recorded to the nearest 0.1 cm. The weight was measured using a wireless Tanita weight scale (BC1000, Tanita Corporation, Tokyo, Japan), connected to a computer. Personal details of the children, including sex, age, and height were entered into the computer, and their body mass index (BMI) was determined. The obtained BMI was converted to percentiles for age, sex, and height and classified as follows: underweight, <5th percentile; normal weight, ≥5th to <85th percentile; overweight, ≥85th to <95th percentile; and obese, ≥95th percentile [29]. The waist circumference (WC), mid-upper arm circumference (MUAC), neck circumference (NC), ankle circumference (AC), calf circumference (CC), and thigh circumference (TC) were measured using an anthropometric tape in centimeters (cm). The waist-to-height ratio (WHtR) was calculated from the waist circumference and height, and a cut-off value of 0.5 was used to classify obesity as previously reported [30].

### 2.5. Blood Pressure Measurements

Blood pressure (BP) was measured using an automated sphygmomanometer (Omron M500, HEM-7321-D, Omron Corporation, Kyoto, Japan). After resting for 5 min, a paediatric cuff attached to the sphygmomanometer was fitted to the bare upper-left arm of children, seated in the upright position with the left arm on the table. Three BP readings, which included the systolic blood pressure (SBP), diastolic blood pressure (DBP), and heart rate (HR), were taken at 2-min intervals. The average BP reading was then computed. BP readings were converted to BP percentiles [31]. Participants were classified as normotensive (SBP and DBP < 90th percentile), pre-hypertensive (SBP and/or DBP ≥ 90th to < 95th percentile), or hypertensive (SBP and/ or DBP ≥ 95th percentile). The mean arterial pressure (MAP) was calculated using this formula:

MAP = (SBP + (2 × DBP)) ÷ 3.(1)

### 2.6. Assessment of Endothelial Function 

The Vicorder device (SMT medical GmbH & Co. KG, Germany) was used to investigate changes in arterial stiffness in children. Participants were asked to lie in a supine position for 10 min before measurements were performed. A standard 10 cm pressure cuff was placed on the upper-right thigh, as high as possible towards the crotch, while a 7 cm pressure cuff was wrapped around the wrist of the same arm. The cuff was closed tight enough to assure a good coupling of the cuff to the femoral artery. The right common carotid artery pulse was palpated on the centre between the base of the neck and chin, where a neck band with an attached neck pressure cuff was placed snugly around the neck with the cuff-bladder placed exactly over the palpated carotid artery pulse. Pressure lines were attached to the cuffs, and the pulse wave velocity was determined.

### 2.7. Sample Collection and Biochemical Analysis

Urine was collected from all participants in sterile tubes and used to quantify the following biochemical parameters. Albumin and creatinine were quantified using the Roche Cobas 6000 analyser, while asymmetric dimethyl-arginine (ADMA), and 8-Hydroxy-2′-deoxyguanosine (8-OHdG) were determined using ELISA kits according to the manufacturer’s protocol. A lipid peroxidation assay was performed based on the quantification of thiobarbituric acid-reactive substances (TBARs), as described by Mallick et al. (2007) [32].

### 2.8. Statistical Analysis

Data was analysed using STATA MP, version 14.1. The distribution of the data was tested for normality using the Shapiro–Wilk test. The study population was normally distributed, and thus, parametric tests were used. An independent sample t-test was used to compare mean differences of study parameters by sex. The Pearson correlation was used to evaluate the relationship of CVD risk factors with obesity and hypertension. A 95% confidence interval was taken, and a *p* value ≤ 0.05 was considered significant. 

## 3. Results

### 3.1. General Characteristics of Study Participants

A total of 306 children aged 6–9 years were recruited for the study, among which 171 were girls and 135 were boys. The study population matched for age, height, weight, BMI, WC, AC, CC, and MUAC between boys and girls. The blood pressure measures SBP, DBP, HR, MAP, and PWV were also not significantly different (*p* > 0.05) between girls and boys. Neck circumference, creatinine, and 8-OHdG were significantly higher (*p* < 0.001) in boys than girls, while albumin was significantly higher (*p* < 0.001) in girls than in boys (Table 1). 

### 3.2. Prevalence of Overweight/Obesity and Pre-Hypertension/Hypertension 

Overweight/obesity (19.28%) and pre-hypertension/hypertension (42.16%) were prevalent in children. Pre-hypertension/hypertension prevalence in this study was twice higher than the prevalence of overweight/obesity (Table 2). The prevalence of overweight/obesity by BMI percentiles and WtHR was higher in girls than in boys. Furthermore, pre-hypertension/hypertension was higher in girls than in boys.

### 3.3. Effect of Body Size on Risk Markers of Cardiovascular Diseases in Children

Creatinine level was significantly higher (*p* < 0.05) in overweight/obese children than in those with normal weight. On the other hand, Albumin, ADMA, PWV, TBARS, and 8-OHdG were not significantly different (*p* > 0.05) between normal weight and overweight/obese children (Table 3).

### 3.4. Effect of Blood Pressure Status on Risk Markers of Cardiovascular Diseases in Children

Pre-hypertensive/hypertensive children had significantly (*p* < 0.001) higher creatinine levels than normotensive children (Table 4). However, albumin, ADMA, and PWV were significantly (*p* < 0.001) higher in normotensive children than in pre-hypertensive/hypertensive children. TBARS and 8-OHdG were not significantly (*p* > 0.05) different between normotensive and hypertensive children (Table 4).

### 3.5. Relationship of Risk Factors of Cardiovascular Diseases with Blood Pressure and Anthropometric Measures in Children

Creatinine was significantly positively correlated (*p* < 0.05) with WC and NC, while ADMA was positively correlated (*p* < 0.05) with MUAC. All other risk factors of CVD did not show any significant association (*p* > 0.05) with the obesity and blood pressure indices (Table 5).

## 4. Discussion

Risk factors for CVDs are becoming more prevalent in children [13]. These call for concern as it is known that risk factors for CVDs begin early in life and track into adulthood. These risk factors include hypertension, obesity, dyslipidaemia, endothelial dysfunction, and oxidative stress, among others [13,14]. Among these factors, obesity and hypertension are the commonly associated hallmarks of CVD and have shown to be prevalent in children [26,27]. In this study, the prevalence of overweight/obesity and pre-hypertension/hypertension were 19.28% and (42.3%), respectively. This finding concurs with previous studies which have shown obesity and hypertension to be prevalent in children in South Africa [33], Kenya [34], as well as in other countries across the world [35,36,37]. This suggests that childhood obesity and hypertension are on the rise in South Africa. 

Although obesity and hypertension are becoming more prevalent in children, their influence on other CVD risk factors in children remains unclear. Obesity and hypertension are often associated with CVD risk factors in adults. Obesity promotes the infiltration and activation of macrophages and monocytes into adipose tissue [17]. The accumulated adipocytes promote the release of a low level of pro-inflammatory adiponectin, while macrophages release elevated levels of pro-inflammatory adipokines, such as interleukin-6 (IL-6) and tumor necrosis factor alpha (TNF-a) [17,18]. These adipokines in turn induce the production of reactive oxygen species (ROS), thereby causing oxidative stress [19,20]. In this study, the oxidative stress markers, TBARS and 8-OHdG tended to be higher in obese subjects but not associated with obesity measures. This finding contradicts the findings of Selvaraju et al. [38] in Alabama, who found that 8-OHdG increased significantly in overweight children as compared to normal-weight children. Our finding suggests that obesity may not be the sole contributor to oxidative stress, as other factors, such as metabolic and physiological processes in the body may result in the generation of ROS, which can induce oxidative damage. 

Also, a low level of adiponectin, which results from obesity, promotes the synthesis of arginase [39]. An elevated level of arginase competes with endothelial nitric oxide synthase (eNOS) for its substrate L-arginine, thereby limiting the production of nitric oxide (NO) [40]. Moreover, eNOS can be inhibited by asymmetric dimethylarginine (ADMA) by competing with its substrate, L-arginine, thereby impairing the production of NO [24,41,42]. Decreased bioavailability of NO results in endothelial dysfunction [43]. A high level of pulse wave velocity (PWV) is indicative of endothelial dysfunction. Although PWV and ADMA levels were insignificantly higher in obese children, MUAC, a marker for obesity, was associated with ADMA, suggesting obesity to possibly promote endothelial dysfunction, as previously confirmed [44].

Studies have shown the association between hypertension and endothelial dysfunction [45]. Though endothelial dysfunction is generally known to cause hypertension as it causes vasoconstriction and arterial stiffness leading to high BP, it has been suggested that high blood pressure and increased flow rate in the blood vessels may affect smooth muscle tone and endothelial function [11,12]. It has also been suggested that increased BP reduces both shear stress and NO synthesis, leading to endothelial dysfunction [11,41]. Findings from this study showed that ADMA and PWV, markers of endothelial function, were significantly higher in normotensive than hypertensive children, suggesting hypertension may not affect endothelial function in children. In adults however, previous studies have indeed confirmed hypertension as the cause of endothelial dysfunction [46,47].

Though hypertension is associated with increased oxidative stress, it remains unclear whether oxidative stress is a result or a cause of hypertension. Oxidative stress has been suggested to cause hypertension as increased ROS may convert NO to more NO-reactive species [21], thereby reducing the NO level, which may lead to endothelial dysfunction. Endothelial dysfunction impairs vasodilation and may possibly promote vasoconstriction, which restricts blood flow and results in increased blood pressure [43]. Also, oxidative stress may promote vascular smooth muscle cell proliferation and hypertrophy, as well as collagen deposition, leading to thickening of the vascular media and narrowing of the vascular lumen [48]. Our findings showed that it may be too early to associate oxidative stress with hypertension in children, though there has been evidence of this association in adult and animal studies [49]. This implies that other sources of ROS generation, such as metabolic and physiological processes other than hypertension may have resulted in oxidative damage observed in this study. 

Certain biomarkers, such as albumin and creatinine which were originally known as early markers of renal disease, have recently been indicated in large epidemiological studies to be independent predictors of CVD in adults [50]. Increased urinary albumin excretion has been associated with increased risk of CVD [51,52]. Also, creatinine has been shown to predict increased risk of CVD [15]. Since obesity and hypertension are major risk factors of CVD, we postulated a possible relationship with albumin and creatinine. A significantly high level of creatinine was observed in obese, as well as hypertensive children. Also, urinary creatinine was positively associated with obesity measures, WC and NC. These findings suggest that obesity and hypertension may affect renal CVD biomarkers. 

This study has provided initial information on the relationships between CVD risk factors, it may not be sufficient to establish a causal relationship of obesity and hypertension with other CVD risk factors, as it was a cross-sectional study. Therefore, these findings may need to be validated in randomized, controlled prospective studies. Epigenetics plays a huge role in the determinants of cardiovascular risk in children; however, the study did not collect data on pregnancy history so as to account for epigenetic determinants. Unhealthy lifestyles, such as consumption of foods rich in calories, fat, and physical inactivity in childhood may also contribute to the development of CVD later in life. The present study did not address epigenetic and lifestyle practices in children which may influence the findings.

## 5. Conclusions

In conclusion, obesity and hypertension were prevalent and found to be associated with renal-cardiovascular risk, while oxidative stress showed a possible association with obesity in 6 to 9 year-old South African children of African descent. The increased prevalence of hypertension and obesity and their association with other risk factors of CVD suggest that South African children of African descent are becoming more prone to developing CVDs. Therefore, early intervention is required for the prevention of CVDs in adolescents in the near future.

## Figures and Tables

**Table 1:** General characteristics of participating children by sex.

Variable	Girls	Boys	*p* Value
N	171	135	
Age (y)	7.79 (8.27–8.09)	8.11 (7.85–8.37)	0.56
HT (cm)	126.95 (124.40–129.50)	128.01 (126.00–130.01)	0.53
WT (kg)	26.64 (25.40–27.88)	28.24 (26.31–30.16)	0.89
BMI (kg/m^2^)	16.34 (15.75–16.93)	16.63 (15.71–17.55)	0.17
NC (cm)	25.39 (25.00–25.79)	26.70 (26.20–27.20)	<0.001
MUAC (cm)	19.91 (19.38–20.45)	19.81 (19.03–20.59)	0.227
WC (cm)	58.71 (57.31–60.12)	59.19 (57.29–61.10)	0.20
CC (cm)	25.64 (24.94–26.33)	26.34 (25.49–27.23)	0.83
AC (cm)	18.66 (18.25–19.10)	19.13 (18.55–19.71)	0.19
SBP (mmHg)	109.46 (107.05–111.86)	108.01 (105.0–111.71)	0.75
DBP (mmHg)	71.27 (69.48–73.07)	69.19 (66.83–71.55)	0.28
HR (bpm)	91.06 (87.74–94.38)	88.65 (92.65)	0.13
MAP (mmHg)	84.00 (82.20–85.80)	82.36 (80.17–84.54)	0.43
Creatinine (mmol/L)	7.74 (6.76–8.70)	9.82 (8.49–11.15)	<0.001
Albumin (mg/L)	44.71 (5.73–83.69)	27.35 (−3.49–58.20)	0.08
TBARS (µM)	0.08 (0.07–0.09)	0.07 (0.06–0.08)	0.94
8-OHdG (ng/mL)	62.73 (58.99–66.48)	65.15 (61.51–68.78)	0.08
ADMA (ng/mL)	73.64 (71.25–76.04)	74.30 (71.22–77.37)	0.22
PWV(m/s)	7.11 (5.54–8.68)	6.18 (5.13–7.23)	0.25

Values were expressed as mean (min CI–max CI); CI: Confidence interval; N: Number of children; HT: Height; WT: Weight; BMI: Body Mass Index; NC: neck circumference; MUAC: mid-upper arm circumference; WC: waist circumference; CC: calf circumference; AC: ankle circumference; SBP: Systolic blood pressure; DBP: Diastolic blood pressure; HR: Heart rate; MAP: Mean arterial pressure; ADMA: Asymmetric dimethylarginine; TBARS: Thiobarbituric acid-reactive substances; 8-OHdG: 8-hydroxyl-2-deoxy-guanosine; PWV: Pulse wave velocity.

**Table 2:** Prevalence of overweight/obesity and pre-hypertension/hypertension among the participating children by cohort and sex.

Variable	Cohort (%)	Girls (%)	Boys (%)
Overweight/obesity by BMI percentiles	59 (19.28)	37 (12.09)	22 (7.19)
Overweight/obesity by WHtR	55 (17.97)	37 (12.09)	18 (5.88)
Pre-hypertension/hypertension	129 (42.16)	74 (24.18)	55 (19.97)

BMI: Body mass index; WHtR: Waist-to-height ratio.

**Table 3:** Effect of obesity on risk factors of cardiovascular diseases in children.

Variable	Normal Weight (95% CI)	Overweight/Obesity (95% CI)	*p* Value
Creatinine (mmol/L)	8.37 (7.45–9.29)	9.52 (8.00–11.05)	0.03
Albumin (mg/L)	43.53 (12.67–74.39)	6.76 (3.72–9.81)	0.16
ADMA (ng/mL)	74.18 (72.19–76.18)	73.32 (68.59–78.06)	0.82
PWV(m/s)	6.69 (5.58–7.80)	7.57 (4.77–10.38)	0.79
TBARS (µM)	0.07 (0.06–0.08)	0.08 (0.07–0.09)	0.23
8-OHdG (ng/mL)	62.90 (60.19–65.62)	69.82 (62.78–76.87)	0.21

Values are expressed as mean (min CI–max CI); CI: Confidence interval; ADMA: Asymmetric dimethylarginine; TBARS: Thiobarbituric acid-reactive substance; 8-OHdG: 8-hydroxyl-2-deoxy-guanosine; PWV: Pulse wave velocity.

**Table 4:** Effect of blood pressure on risk factors of cardiovascular diseases in children.

Variable	Normotensive (95% CI)	Pre-HT/HT (95% CI)	*p* Value
Creatinine (mmol/L)	8.55 (7.4–9.7)	8.63 (7.53–9.72)	<0.001
Albumin (mg/L)	39.03 (6.27–71.78)	32.03 (–4.22–68.28)	<0.001
ADMA (ng/mL)	75.08 (73.25–76.91)	73.07 (69.96–76.19)	<0.001
PWV(m/s)	6.94 (5.41–8.47)	6.41 (5.25–7.56)	0.02
TBARS (µM)	0.07 (0.06–0.08)	0.08 (0.07–0.09)	0.40
8-OHdG (ng/mL)	64.91 (61.26–68.58)	64.78 (60.63–68.94)	0.58

Values are expressed as mean (min CI–max CI); CI: Confidence interval; ADMA: Asymmetric dimethylarginine; TBARS: Thiobarbituric acid-reactive substance; 8-OHdG: 8-hydroxyl-2-deoxy-guanosine; PWV: Pulse wave velocity. Pre-HT/HT: Pre-hypertensive/Hypertensive.

**Table 5:** Relationship between blood pressure parameters and risk factors of cardiovascular diseases in children.

	SBP	DBP	HR	BMI	WC	MUAC	NC
Creatinine (mmol/L)	−0.036	0.004	0.008	0.067	0.189 *	0.072	0.246 *
Albumin (mg/L)	0.029	0.043	0.027	−0.067	0.037	−0.023	−0.086
ADMA (ng/mL)	−0.041	0.016	0.004	0.113	0.104	0.175*	0.102
PWV(m/s)	0.09	0.22	0.02	−0.006	−0.022	0.053	0.003
TBAR (µM)	0.001	0.136	0.032	−0.038	0.064	−0.059	0.132
8-OHdG (ng/mL)	0.041	0.011	−0.031	0.034	0.034	0.031	0.102

Values are expressed as mean (min CI–max CI); * indicates a significant relationship where *p* < 0.05; BMI: Body mass index; WC: Waist circumference; NC: Neck circumference; MUAC: Mid-upper arm circumference; ADMA: Asymmetric dimethylarginine; TBARS: Thiobarbituric acid-reactive substance; 8-OHdG: 8-hydroxyl-2-deoxy-guanosine; PWV: Pulse wave velocity.
